# Genetic Map Construction and Detection of Genetic Loci Underlying Segregation Distortion in an Intraspecific Cross of *Populus deltoides*

**DOI:** 10.1371/journal.pone.0126077

**Published:** 2015-05-05

**Authors:** Wencai Zhou, Zaixiang Tang, Jing Hou, Nan Hu, Tongming Yin

**Affiliations:** 1 Co-Innovation Center for Sustainable Forestry in Southern China, College of Forestry, Nanjing Forestry University, Nanjing 210037, China; 2 Department of Epidemiology and Biostatistics, School of Public Health, Medical College of Soochow University, Suzhou 215123, China; 3 Jiangxi Academy of Forestry, Nanchang 330032, China; Key Laboratory of Horticultural Plant Biology (MOE), CHINA

## Abstract

Based on a two-way pseudo-testcross strategy, high density and complete coverage linkage maps were constructed for the maternal and paternal parents of an intraspecific F2 pedigree of *Populus deltoides*. A total of 1,107 testcross markers were obtained, and the mapping population consisted of 376 progeny. Among these markers, 597 were from the mother, and were assigned into 19 linkage groups, spanning a total genetic distance of 1,940.3 cM. The remaining 519 markers were from the father, and were also were mapped into 19 linkage groups, covering 2,496.3 cM. The genome coverage of both maps was estimated as greater than 99.9% at 20 cM per marker, and the numbers of linkage groups of both maps were in accordance with the 19 haploid chromosomes in *Populus*. Marker segregation distortion was observed in large contiguous blocks on some of the linkage groups. Subsequently, we mapped the segregation distortion loci in this mapping pedigree. Altogether, eight segregation distortion loci with significant logarithm of odds supports were detected. Segregation distortion indicated the uneven transmission of the alternate alleles from the mapping parents. The corresponding genome regions might contain deleterious genes or be associated with hybridization incompatibility. In addition to the detection of segregation distortion loci, the established genetic maps will serve as a basic resource for mapping genetic loci controlling traits of interest in future studies.

## Introduction

Genetic linkage maps provide unique tools in breeding and genomic studies in a variety of ways. Based on genetic linkage maps, markers tightly linked to traits of interest are useful for quantitative trait locus (QTL) detection, marker-aided selection, and marker-assisted cloning [1–3]. Genetic maps are also powerful tools to study genetic mechanisms triggering the uneven transmission of genetic materials in many different plant species [4–6]. At the population level, fitness and other evolutionary agents can be reflected by the variation of gene frequencies [7, 8]. As a result, the interactions of differentiated genes and genomes in a hybrid genetic background can be revealed, to some extent, by information of the gene frequencies on linkage maps [4].

Segregation distortion is a common phenomenon that has been observed in many mapping studies [9–13]. The genetic mechanism triggering segregation distortion has attracted the attentions of evolutionary biologists. Vogl and Xu [14] proposed that these deviations were caused by viability selection occurring at loci linked to the markers with distorted inheritance. These loci are referred to as segregation distortion loci (SDLs). An SDL is hidden, but has an important function in evolution by controlling the viability of individuals with different genotypes of the locus [15]; hence, mapping SDLs is significant to restrict the target genomic regions for further exploration at the molecular level [16]. Many methods have been developed to map SDLs [8, 14, 17–20]. Recently, Luo et al. [21] proposed a consensus model for mapping SDLs using the allelic frequencies of markers along the linkage groups. This analytical method is more user friendly because of the integration of the SDL mapping module into the PROC QTL software [22].

The genus *Populus* has been widely used as a model system in tree genomic studies because of its fast growth, wide distribution, amenability for gene transformation, ease of vegetative propagation, and small genome size (ca. 480 Mb) [23–26]. Since the construction of the first linkage map for aspen [27], a number of maps for different poplars have been developed [28–35]. These maps were widely used for different perspectives, including QTL detection [36–38], comparative mapping [30, 39], and genome assembly [40]. Marker segregation distortion is a common phenomenon observed during map construction, which can be used to infer the genomic regions associated with gamete selection in the progeny of a particular hybridization. In previous studies, maker segregation distortion was explored in several *Populus* species [30, 32, 41, 42]. However, SDL detection based on a specially designed statistical model, has not been conducted in any poplar species thus far. In this study, we constructed high-density linkage maps using a large number of progeny of an intraspecific cross of *Populus deltoides*. Based on the established maps, we detected those SDLs that triggered segregation distortion of the mapped markers. In addition to SDL detection, the maps developed in this study will provide a useful resource to localize genes controlling traits of interest in future studies.

## Materials and Method

### Plant material and DNA preparation

The mapping pedigree in this study was an intraspecific F_2_ cross, which comprised 3,186 offspring. The grandmother and the grandfather of this pedigree were *P*. *deltoides* clone “T-120” and “I-63”, respectively. “T-120” was introduced from Texas, USA, and “I-63” was introduced from Italy. The mother and father of this pedigree are siblings from “T-120”×“I-63”, they were named as “clone 2–2” and “clone 2–38”. The mapping population was maintained at the poplar plantation farm in Sihong county of Jiangsu Province, China. From this mapping pedigree, 384 progeny were randomly selected for the mapping study. Young leaves were collected from each individual, and genomic DNA was extracted by using the DNeasy plant kit (Qiagen, Helden, Germany). DNA quality and quantity were assessed using a NanoDrop 2000 (Thermo Scientific, MA, USA) and by electrophoresis on 1% agarose gels. The field studies did not involve any endangered or protected species, and a sample collection was authorized by the administration office of the poplar plantation farm in Sihong county of Jiangsu Province, China.

### Amplified fragment length polymorphism (AFLP) genotyping and marker nomenclature

AFLP genotyping was performed following the protocol described by Vos et al. [43]. First, about 400 ng of *Populus* DNA was digested with restriction enzymes of *Eco*RI and *Mse*I, followed by ligation of *Eco*RI and *Mse*I adapters. The ligation mixture was diluted 1:10 with deionized water. Subsequently, pre-amplification was performed using an *Eco*RI (*E*) primer with no selective nucleotides and an *Mse*I (*M*) primer with a “C” or “T” selective nucleotide. The reaction volume for pre-amplification was 25 μL, containing 3 μL diluted ligation mixture, 1.0 U Taq polymerase, 2.5 μL 10× buffer (100 μM Tris-HCl, pH 8.3, 500 mM KCl, 20 Mm MgCl_2_), 5 ng of the *E*-primer, 30 ng of the *M*-primer, 0.1 g/L BSA, and 0.2 mM dNTPs. PCR amplifications were performed for 20 cycles of 94°C for 30 s, 56°C for 30 s, and 72°C for 120 s. Finally, selective amplification was performed in a volume of 15 μL, containing 3 μL of 1:15 dilutions of the pre-amplification products, 3 ng of the *E*-primer with two selective nucleotides, and 10 ng of the *M*-primer with three selective nucleotides (5-FAM labeled), 1.0 U Taq polymerase, 1.5 μL 10× buffer (100 μM Tris-HCl, pH 8.3, 500 mM KCl, 20 Mm MgCl_2_), 0.2 mM dNTPs, 1% (v/v) deionized formamide, and 0.1 g/L BSA. PCR amplification was performed for 12 touchdown cycles of 94°C for 30 s, 65°C for 30 s (reduced by 0.7°C/cycle), and 72°C for 120 s; followed by 23 cycles of 94°C for 30 s, 56°C for 30 s, and 72°C for 120 s.

**Fig 1:**
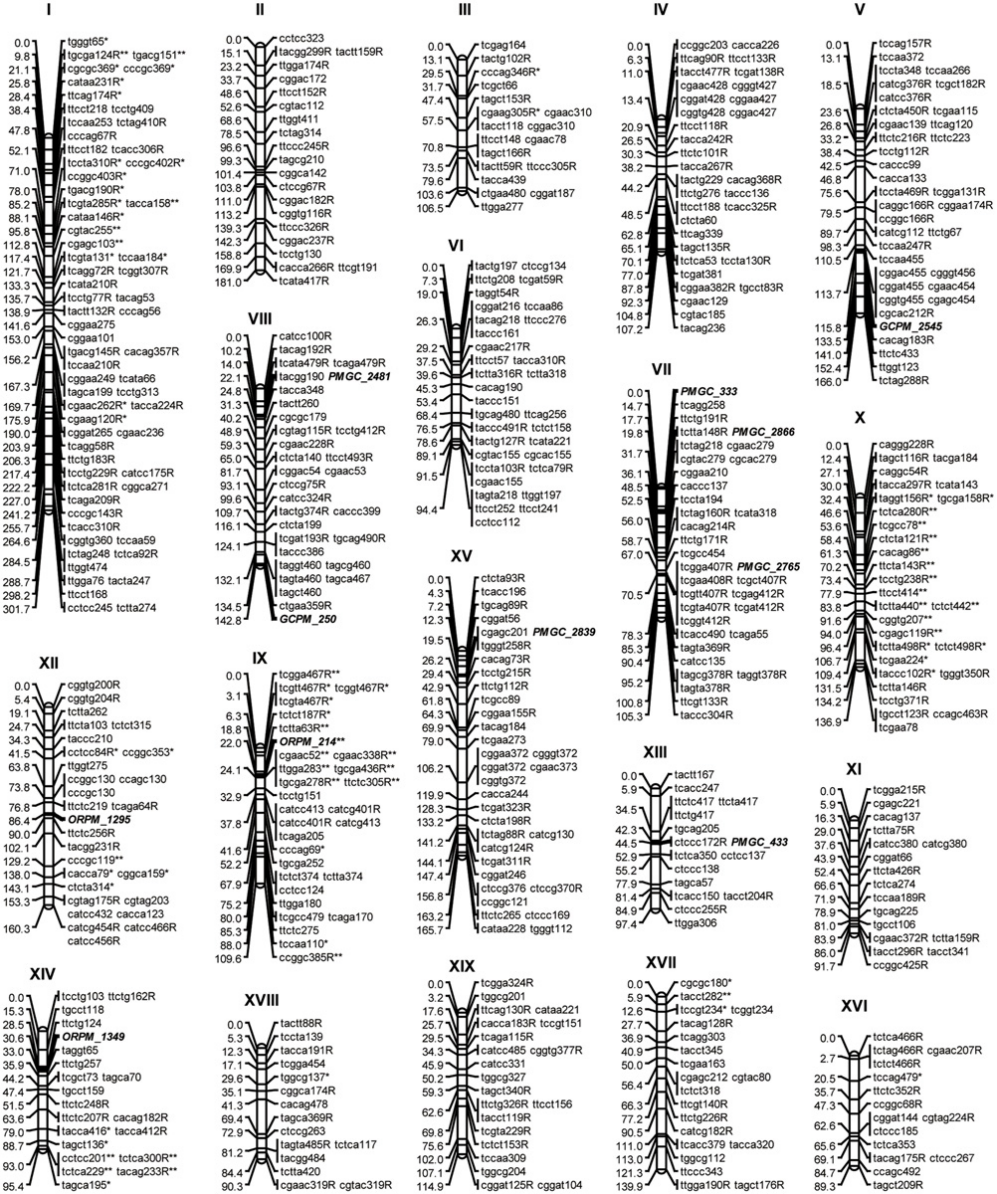
Genetic linkage map of the male parent. Note: Markers in bold and italic fonts are SSR markers; for marker ending with a letter of “R”, the corresponding marker is repulsion linkage phase; markers with significant segregation distortion are indicated with “*” when *P*≤0.05, or “**” when *P*≤0.01.

PCR products were analyzed on an ABI 3730 capillary sequencer (Applied Biosystems, CA, USA) using the standard genotyping module and were scored with the GeneMapper v3.7 software (Applied Biosystems, CA, USA). Fragments ranging from 50 to 500 base pairs were scored. The AFLP markers were named using a combination of letters and digital numbers: the first three letters were the selective nucleotides of the M-primer, the last two letters were the selective nucleotides of the E-primer, and the digital number was the allele size in base pairs (Fig 1). Genotyping with the two parents and six progeny allowed us to select 84 AFLP primer combinations (S1 Table), which generated highly polymorphic and clearly segregated peaks from a total of 256 primer pairs. The selected primer combinations were used for genotyping the mapping population.

### Simple sequence repeat (SSR) markers analysis

Anueploids and polyploids are occasionally observed in the hybrids of poplar species [32], which lead to systematic errors in estimating the recombination ratio. To exclude the anueploids and polyploids from the mapping population, 12 fully informative SSR primer pairs (S2 Table) were screened from the primer resources of the Oak Ridge National Laboratory (PMGC_, GCPM_ and ORNL_ prefix [44, 45]) to detect individuals that had extra alleles or lacked the expected alleles. SSR genotyping was performed in a volume of 15 μL containing 30 ng of genomic DNA as template, 20 ng of both forward and reverse primers, 0.2 mM of each dNTP, 0.01 μL Fluorescein-12-dUTP (1 mM; Roche Diagnostics, IN, USA), 1 U Taq DNA polymerase, 1.5 μL 10× buffer (100 μM Tris-HCl, pH 8.3, 500 mM KCl, 20 mM MgCl_2_), and 0.1 g/L BSA. PCR reactions were performed using the following profile: 94°C for 4 min; 30 cycles of 94°C for 30 s, 56°C for 30 s, 72°C for 30 s; and a final extension of 94°C for 10 min. The PCR products were analyzed on an ABI 3730 capillary sequencer (Applied Biosystems, CA, USA) using the standard genotyping module, following the same procedure as that for AFLP analysis.

### Map construction

The linkage maps were constructed using MapMaker v3.0 [46], following the two-way pseudo-testcross mapping strategy [47, 48]. The maternal and paternal segregated testcross markers were analyzed separately for each parent. During data preparation, the AFLP dataset was duplicated to allow the MapMaker software to detect repulsion-phase linkages. Linkage groups were assigned under a logarithm of odds (LOD) score of 10.0 and a maximal recombination fraction (θ) of 0.30. For each linkage group, markers were ordered using the “Order” command with a default LOD of 3.0, the best order of markers was then confirmed using the “Ripple” command. In each linkage group, tightly linked markers (≤ 2 cM) were placed in the same marker bin. Linkage maps were generated with the ‘Map’ command in Kosambi distance. The map charts were drawn using MapChart v2.1 [49].

Genome coverage was estimated according to the function given by Lange and Boehnke [50], assuming a random marker distribution, as follows: *C* = 1 − *e* ^−2*md*/*G*_*e*_^, where *C* is the coverage of the genome within *d* cM of a marker, *m* is the total number of markers, and *G*_*e*_ is the estimated genome length. In this study, the coverage estimation was performed with *d* = 20 cM.

### Segregation distortion analysis

A χ^2^ test was performed for each marker to assess whether it significantly deviated from Mendelian segregation ratios (1:1). Segregation distorted markers were indicated with a “*” at a significance of *P*≤0.05, or a “**” at a significance of *P*≤0.01 (Fig 1).

### Marker distribution analysis

Marker distribution throughout different linkage groups was evaluated using the method described by Remington et al. [51], which examines whether markers are clustered or dispersed on each linkage group by testing the expected number of markers (*λ*_i_) under the assumption of an even distribution of markers: λ_i_ = *mL_i_*/∑*L_i_* where *m* was the total number of markers, and *L*_*i*_ was the observed map length plus two times the size of the average interval of linkage group *i*. The probabilities that a linkage group contained more or fewer markers than the expected number (*λ*_i_) were evaluated using a two-tailed cumulative Poisson calculator. Subsequently, the markers clustered or dispersed in regions within each linkage group were evaluated following the method described by Yin et al. [32].

### Detection of SDLs

Based on the established genetic maps, SDL detection was performed using PROC QTL v2.0 (http://statgen.ucr.edu/download/software) [22], with a step length of 1 cM along each linkage group. In the output results table, the threshold to call a significant SDL was set at LOD ≥ 2.0.

### Data access

The genotyping data generated in this study were deposited at website, http://dx.doi.org/10.6084/m9.figshare.1363641.

## Results

### Marker polymorphism and segregation

The informativeness of the SSR primer pairs is listed in S2 Table. With these fully informative SSR markers, all progeny are expected to inherit an allele from the mother and a complementary allele from the father at each of the loci. However, eight progeny were observed either to possess extra alleles or be hemizygous, or were detected to have a non-parental allele at more than one of these SSR loci, indicating they were either polyploids/anueploids or resulted from pollen contamination; hence, the eight individuals were excluded from the mapping population. As a result, 376 progeny were finally included in the linkage map construction. Genotyping with 84 AFLP primer combinations generated 1,487 segregating loci **(** S1 Table **)** for this mapping pedigree. Among these, 587 (39.48%) were testcross markers from the female parent, 508 (34.16%) were testcross markers from the male parent, and 392 (26.36%) were intercross markers. The number of segregating markers varied among different primer combinations, ranging from 3 to 39 segregating bands per primer pair.

### Map construction

Poplars are outbreeding plants, and the heterozygosity or homozygosity of markers varied among different genetic loci of the poplar genomes. In this study, most of the segregating markers are testcross markers, which means they are heterozygous in one of the parents and are recessively homologous (possess double null alleles) in the other parent. Besides, a relatively high number of intercross markers (heterozygous in both parents) were also generated. However, the intercross markers provided limited information to estimate the recombination frequency precisely [52], and thus they were excluded from map construction. Following the two-way pseudo-testcross mapping strategy [47, 48], two genetic maps, one for each of the parents, were constructed. During the map construction, the markers with significant departures from Mendelian segregation were not excluded because of their possible relation to the genes underlying traits of interest [28, 30, 32]. For the paternal map, 508 AFLPs and 11 paternally heterozygous SSRs were assigned to 19 linkage groups, covering a total genetic distance of 2,496.3 cM (Fig 1). On this map, the linkage group sizes ranged from 89.3 cM to 301.7 cM, and the mapped markers were allocated into 327 marker bins. The average distance between adjacent marker bins was 7.63 cM. For the maternal map, 587 AFLPs, together with 10 maternally heterozygous SSRs, were also assigned into 19 linkage groups, covering a total genetic length of 1,940.3 cM. The linkage groups on the female map ranged from 50 cM to 224.2 cM in size, and the mapped markers on the maternal map were distributed in 308 marker bins (S1 Fig). The average distance between adjacent marker bins on the female map was 6.30 cM. Using the function given by Lange and Boehnke (1982), the coverages of the established map were estimated as 99.94% and 99.99% of the male and female genome at 20 cM per marker, respectively.

### Marker distribution

Statistics on marker distribution revealed that there was a significant excess of markers on linkage groups IV, VI, and VII on the male map (*P*≤0.025), and significantly less than the expected number of markers on linkage group II (Table 1). Further analysis of marker distribution within each linkage group showed the presence of significant marker clusters in all of the linkage groups. These marker clusters comprised 195 markers, accounting for 38.39% of all markers, but covered only 6.17% of the map distance. Marker dispersion regions were also observed on eight of the linkage groups, which contained 5.91% of the mapped markers, while spanning 15.68% of the map distance. On the maternal map, linkage groups I, X, and XIX contained significantly more than the expected number of markers, and linkage group XII had significantly fewer than the expected number of markers (S3 Table). Analysis of marker distribution within each linkage group on the maternal map also revealed regions with an overabundance of markers on all of the linkage groups. These regions comprised 271 markers, accounting for 46.17% of the mapped markers, but covered only 5.05% of the total map distance of the maternal map. Furthermore, we also detected marker dispersion regions on 12 of the linkage groups, which comprised 9.03% of the mapped markers, while spanning 26.81% of the total map length.

**Table 1:** AFLP marker distribution on the paternal map.

Linkage group	The observed map length (cM)	The expected map length (cM)	The expected number of AFLPs	The observed number of AFLPs	Poisson two-tailed *P*-value
I	301.7	311.13	58.47	65	0.2128
II	181	199.1	37.42	21	0.0026**
III	106.5	119.03	22.37	18	0.2098
IV	107.2	113.9	21.41	33	0.0119*
V	166	175.49	32.98	36	0.3220
VI	94.4	100.3	18.85	33	0.0020**
VII	105.3	112.09	21.07	32	0.0158*
VIII	142.8	153	28.75	29	0.5065
IX	109.6	117.43	22.07	29	0.0896
X	136.9	146.68	27.57	29	0.4174
XI	91.7	103.16	19.39	17	0.3456
XII	160.3	172.63	32.44	27	0.1945
XIII	97.4	111.31	20.92	15	0.1143
XIV	95.4	105.44	19.82	20	0.5134
XV	165.7	176.06	33.09	33	0.5292
XVI	89.3	102.06	19.18	15	0.8593
XVII	139.9	154.63	29.06	20	0.0500
XVIII	90.3	103.2	19.4	15	0.1902
XIX	114.9	126.39	23.75	21	0.3318
Total	2496.3	2703.02	508	508	

Note:

“*” indicates significance at α = 0.05;

“**” indicates significance at α = 0.01; the probabilities for clustering or dispersal of AFLP markers(m_*i*_≥*λ*_i_ or m_*i*_≤*λ*_i_) were evaluated by using a two-tailed cumulative Poisson calculator (*P* ≤ 0.025 is significant at α = 0.05).

### Segregation distortion and SDLs detection

Of the 519 markers mapped on the male map, 73 (14.07%) were significantly distorted from the expected 1:1 segregation ratio (α ≤ 0.05), and most of the segregation distorted markers (86.30%) were observed on linkage groups I, IX, X, XII, and XIV (Fig 1, S4 Table). On these linkage groups, large contiguous blocks with segregation-distorted markers were detected. For instance, the segregation-distorted clusters spanned a region of 89 cM (29.58% of the group length) and 78.2 cM (57.12% of the group length) on linkage groups IX and X, respectively. Whereas on the female map, 28 markers (4.69% of the mapped markers) were detected to skew significantly from the 1:1 segregation ratio (α ≤ 0.05). These markers were scattered over 11 of the linkage groups, and only one large segregation distorted cluster was found at the peritelomeric end of linkage group III (S1 Fig, S5 Table).

**Fig 2:**
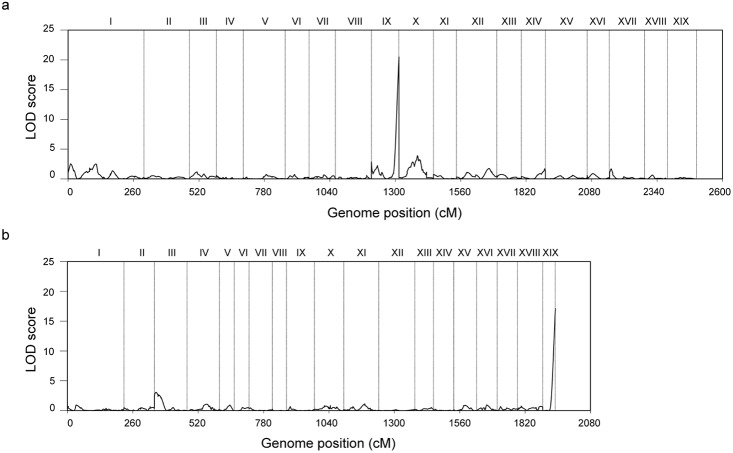
Profiles of the LOD score for SDL declaration throughout the male and female maps. 2a. The male map; 2b. The female map. Note: Y-axis shows the LOD score, and X-axis displays the accumulated genetic length in order of the linkage group numbers. The Rome digital on the top of the figure are the linkage group identities.

SDLs analysis detected six and two SDLs with significant LOD support on the paternal and maternal maps, respectively (Table 2, Fig 2). Each of the detected SDLs caused marker segregation distortion over a larger genetic distance (Table 2). Segregation distortion reflected the uneven descent of alleles on the alternate chromatids from the parents. For a particular AFLP marker, a gamete either inherits the visible allele or the invisible allele. Notably, segregation distorted markers caused by a specific SDL tended to be linked in coupling phase. For some of the SDLs, the visible alleles of the affected markers dominated the offspring, while for some other SDLs, the invisible alleles (null alleles) of the affected markers had a higher chance to descend to the progeny (Table 2).

**Table 2:** The SDLs detected based on the established genetic maps.

SDL	Genetic map	Linkage group	Peak position(cM)	LOD score	Maximum χ^2^ -value	Effect span(cM)	Dominant allele
1	Paternal	I	9.82	2.53	11.33	33.4	-
2	Paternal	I	109.8	2.49	11.39	58	+
3	Paternal	IX	0	2.81	21.88	14.5	+
4	Paternal	IX	24.1	2.23	9.98	14	+
5	Paternal	IX	109.6	20.41	89.78	23	-
6	Paternal	X	73.4	3.91	17.11	78.2	+
7	Maternal	III	6.7	3.06	13.51	40.8	-
8	Maternal	XIX	50	17.11	83.64	12.2	-

Note: Effect span indicates the genetic length that a SDL would cause segregation distortion of makers within the region. “+” represents segregation distortion skews to more visible alleles for markers in the affected genome region, and “-” indicates segregation distortion skews to less visible alleles for markers in the affected genome region.

## Discussion

The AFLP technique generates multiple markers simultaneously. Referring to the presence of neighboring peaks, null alleles can be discriminated from failed amplification. Taking together the large number of progeny used for map construction, the quality of the established maps is highly ensured. In this study, a large number of testcross markers were generated for each of the mapping parents. As a result, we obtained male and female maps both with 19 linkage group numbers, which equals the number of haploid chromosomes in poplar. The established genetic maps were estimated to achieve nearly complete coverage of the poplar genome.

High heterogeneities in marker density were revealed by examining the marker distribution, and substantial marker clusters were observed on both the paternal and maternal maps. These marker clusters might have resulted from either the overabundance of restriction sites of *Eco*RI and *Mse*I, or from recombination suppression in the corresponding genomic regions [53, 54]. Notably, the observed length of the male map was longer than that of the female map, which is in accordance with some of the previous mapping studies on poplar species [39, 55]. However, this observation is inconsistent with the general tendency that higher recombination rates occur in female angiosperm plants [56]. Dioecy of angiosperm plants are more often controlled by a heterogametic male system, while species with female heterogametes are less common [57, 58]. Poplars are dioecious plants, and mapping studies on *Populus deltoides* [40] and *Populus alba* [39] revealed that their sex determination occurred through a ZW system, in which the female plant was the heterogametic sex, although the XY (male heterogamety) sex-determining system could also be present in some other members of the genus *Populus* [33, 59]. The poplar species involved in this study is *P*. *deltoides*, thus we propose that the difference in the genetic lengths of the male and female maps is associated with the sex-determining system of this poplar species. However, this hypothesis needs to be tested with more mapping data in different poplar species.

Segregation distorted markers have been frequently observed in mapping studies in many organisms, and are also a common in poplars [32, 33, 39]. In the present study, the proportion of segregation-distorted markers was relatively high, especially for the male parent. Segregation distorted markers may link to genes or traits of interest [30, 32]. Thus, the exclusion of such markers could bias the data and result in the loss of some important information. In this study, we included all of the segregation-distorted markers for map construction. Segregation distortion might be associated with certain non-biological factors, such as finite sampling, sampling errors, and genotyping errors [13, 60]. In this study, we genotyped a large number of progeny using a highly reliable marker technique. In particular, the observation of continuous segregation distorted blocks suggested that their occurrence could not be explained by non-biological factors alone [16, 61]. By contrast, biological factors are more likely to be the ultimate causes of the segregation distortion observed in this study. SDL mapping based on the assumption of viability selection [21, 62] is a powerful means for uncovering the hidden genetic biological factors. Viability selection might be associated with meiotic drive, which favors the transmission of one allele over the other [63, 64]. In addition, hybrid incompatibility is also a key biological factor causing uneven transmission of alternate alleles, which is frequently caused by disrupted genetic interactions among loci of parental lineages [65], resulting in the nonrandom elimination of particular allelic combinations. For outbred species such as poplars, inbreeding depression is another critically mechanism underlying segregation distortion. The mapping population used in this study is an inbred poplar pedigree. Inbreeding would increase the rates of homozygosity of deleterious recessive genes [9, 41, 66, 67], resulting in reduced survival of the homozygotes compared with the heterozygotes, leading to the occurrence of segregation distortion [68].

In this study, markers from the male parent had a higher segregation distortion ratio than those from the female parent. According to Haldane’s rule, the heterogametic sex is more likely to be sterile or rare in the hybrids [69]. Sex determination of *P*. *deltoides* occurred through a ZW system in which the female plant was the heterogametic sex [40]. Early empirical observations in several *Populus* species revealed a preponderance of male plants, especially under the harsh environment [70]. Male surplus was also observed in several full-sib mapping pedigrees of different poplar species [39, 40, 59]. However, the progeny of this mapping pedigree is still at the juvenile stage, and require several years to arrive at sexual maturation. At the current developmental stage, it remains unclear whether the occurrence of segregation distortion is associated with sex differentiation of the progeny or not.

QTL mapping based on phenotypic data provides an effective tool to dissect the genetic mechanisms underlying fitness traits [71–73]. However, the occurrence of marker segregation distortion is caused by the elimination of particular individuals, and the phenotypic data of the eliminated individuals are unavailable. As an alternate approach, SDL detection is independent from the phenotypic data, which enabled us to map the genetic loci underlying marker segregation distortion based on the information of allele frequencies. Although the exact genetic factors cannot be completely resolved merely based on SDL analysis; nevertheless, the detected SDLs provided essential information to explore the biological factors underlying the uneven transmission of gametes from the mapping parents in future studies.

## Conclusions

In this study, we constructed highly dense genetic maps for the mapping parents of an F_2_ full-sib family of *P*. *deltoides*. Large contiguous blocks of marker segregation distortion were observed on some of the linkage groups, and eight SDLs were detected. The detected SDLs provide essential information for further dissection of the biological factors underlying the uneven descent of gametes from the mapping parents. Meanwhile, an ongoing project using single nucleotide polymorphism markers to enhance the informativeness of the present maps will ultimately enable us to align the established maps to the poplar genome sequence. The maps developed in this study will serve as a basic platform to uncover genes controlling many other traits of interest in the future.

## Supporting Information

S1 Fig Genetic linkage map of the female parent. Note: Markers in bold and italic fonts are SSR markers; for marker ending with a letter of “R”, the corresponding marker is repulsion linkage phase; markers with significant segregation distortion are indicated with “*” when *P*≤0.05, or “**” when *P*≤0.01.
(TIF)

S1 Table Number of segregating markers generated with different AFLP primer combinations. (DOCX)

S2 Table The zygosity of the mapping parents revealed by each of the fully-informative SSR marker. (DOCX)

S3 Table AFLP Marker distribution on the maternal map. Note: “*” indicates significance at α = 0.05; “**” indicates significance at α = 0.01; the probabilities for clustering or dispersal of AFLP markers(m_*i*_≥*λ*_i_ or m_*i*_≤*λ*_i_) were evaluated by using a two-tailed cumulative Poisson calculator (*P* ≤ 0.025 is significant at α = 0.05).
(DOCX)

S4 Table Statistics of the distorted markers on the male map. (DOCX)

S5 Table Statistics of the distorted markers on the female map. (DOCX)
