# Supplementary figures and images for: Genetic Map Construction and Detection of Genetic Loci Underlying Segregation Distortion in an Intraspecific Cross of Populus deltoides
Source: PLoS One. 2015 May 5;10(5):e0126077. doi: 10.1371/journal.pone.0126077 (PMC4420497; doi:10.1371/journal.pone.0126077)

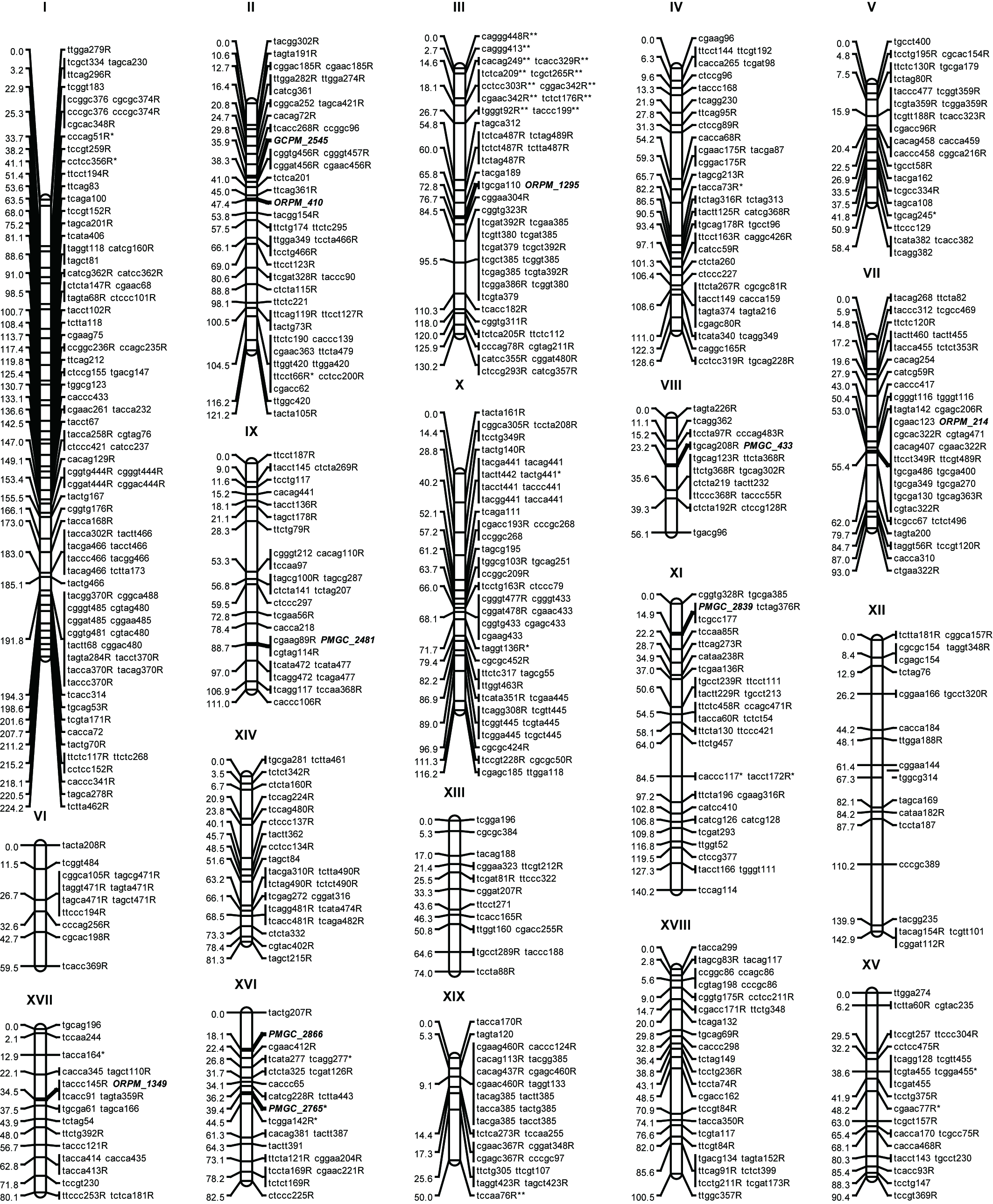

Supplement: S1 Fig — Note: Markers in bold and italic fonts are SSR markers; for marker ending with a letter of “R”, the corresponding marker is repulsion linkage phase; markers with significant segregation distortion are indicated with “*” when P≤0.05, or “**” when P≤0.01. (TIF) [file pone.0126077.s001.tif]
