# Supplementary material for: Genetic Map Construction and Detection of Genetic Loci Underlying Segregation Distortion in an Intraspecific Cross of Populus deltoides
Source: PLoS One. 2015 May 5;10(5):e0126077. doi: 10.1371/journal.pone.0126077 (PMC4420497; doi:10.1371/journal.pone.0126077)
Supplement: S1 Table — (DOCX) [file pone.0126077.s002.docx]

Table S1.

| ***E-*primer** | ***M-*primer** | **Number of segregated bands** | **Size range (bp)** |
| --- | --- | --- | --- |
| E-aa | M-cgg | 16 | 101-485 |
|  | M-ctg | 3 | 322-480 |
|  | M-tcc | 24 | 59-455 |
|  | M-tcg | 17 | 55-445 |
|  | M-cat | 6 | 146-238 |
| E-ac | M-cgg | 18 | 54-480 |
|  | M-cgc | 9 | 154-348 |
|  | M-cgt | 14 | 75-480 |
|  | M-cga | 41 | 52-460 |
| E-ag | M-ccc | 11 | 51-483 |
|  | M-cga | 11 | 75-460 |
|  | M-tac | 24 | 53-494 |
|  | M-tcc | 9 | 52-481 |
|  | M-tgc | 23 | 53-491 |
|  | M-cac | 27 | 72-478 |
|  | M-ttc | 22 | 83-476 |
|  | M-tct | 25 | 54-490 |
|  | M-tcg | 5 | 164-465 |
|  | M-cgt | 15 | 76-480 |
| E-at | M-tcg | 25 | 59-455 |
|  | M-cgg | 23 | 56-485 |
| E-ca | M-tac | 39 | 60-495 |
|  | M-tag | 21 | 57-471 |
|  | M-cac | 25 | 68-468 |
|  | M-tct | 22 | 53-487 |
|  | M-cgg | 12 | 105-488 |
| E-cc | M-cga | 8 | 62-255 |
|  | M-cat | 21 | 59-485 |
|  | M-cct | 23 | 64-476 |
|  | M-ctc | 18 | 78-421 |
|  | M-tac | 29 | 55-494 |
|  | M-tcg | 13 | 67-479 |
|  | M-ttc | 17 | 129-492 |
|  | M-tca | 23 | 91-490 |
|  | M-cac | 18 | 65-458 |
| E-cg | M-tga | 7 | 96-190 |
|  | M-tgg | 12 | 103-432 |
|  | M-cat | 22 | 59-456 |
|  | M-ctc | 15 | 67-489 |
|  | M-tag | 13 | 55-471 |
| E-ct | M-tag | 22 | 72-471 |
|  | M-tac | 27 | 67-494 |
|  | M-tcg | 16 | 66-450 |
|  | M-tct | 23 | 54-498 |
|  | M-tgc | 20 | 58-400 |
|  | M-ttc | 35 | 57-493 |
| E-ga | M-tac | 15 | 78-494 |
|  | M-tca | 20 | 55-482 |
|  | M-tcg | 16 | 131-467 |
|  | M-tgc | 21 | 61-486 |
|  | M-ttg | 23 | 76-437 |
| E-gc | M-ccg | 15 | 68-464 |
|  | M-cga | 16 | 80-460 |
|  | M-ccc | 12 | 86-402 |
|  | M-ttg | 7 | 93-436 |
|  | M-cag | 9 | 54-426 |
|  | M-cgc | 13 | 50-461 |
|  | M-cca | 8 | 86-492 |
| E-gg | M-tca | 16 | 58-481 |
|  | M-cag | 7 | 76-453 |
|  | M-tac | 16 | 106-494 |
| E-gt | M-ttc | 16 | 84-489 |
|  | M-tct | 8 | 60-498 |
|  | M-tcc | 12 | 84-369 |
|  | M-tcg | 12 | 183-484 |
|  | M-ttg | 13 | 52-474 |
|  | M-tgg | 8 | 63-350 |
|  | M-tag | 14 | 54-471 |
|  | M-cgg | 13 | 56-485 |
| E-ta | M-tac | 12 | 68-494 |
|  | M-tag | 24 | 68-485 |
|  | M-ctc | 28 | 60-489 |
|  | M-tct | 26 | 59-498 |
|  | M-tcg | 15 | 103-467 |
|  | M-tcc | 16 | 74-469 |
|  | M-ttc | 16 | 82-479 |
|  | M-tca | 16 | 66-479 |
| E-tc | M-ttc | 28 | 101-458 |
| E-tg | M-ttc | 30 | 67-466 |
|  | M-tcc | 26 | 59-466 |
|  | M-tac | 16 | 70-494 |
|  | M-cgg | 23 | 75-481 |
| E-tt | M-tac | 25 | 59-494 |
|  | M-tcg | 7 | 101-467 |
| total |  | 1487 |  |
