# Supplementary material for: Genetic Map Construction and Detection of Genetic Loci Underlying Segregation Distortion in an Intraspecific Cross of Populus deltoides
Source: PLoS One. 2015 May 5;10(5):e0126077. doi: 10.1371/journal.pone.0126077 (PMC4420497; doi:10.1371/journal.pone.0126077)
Supplement: S2 Table — (DOCX) [file pone.0126077.s003.docx]

Table S2.

| Primer  name | Forward primer sequence  (5’- 3’) | Reverse primer sequence  (5’-3’) | Zygosity in the maternal parent | Zygosity in the paternal parent |
| --- | --- | --- | --- | --- |
| GCPM_2545 | TCTCTCCTGGTAAGTAAGTCTGT | CAGCATGTTTCTTCAGTCAA | Heterozygous | Heterozygous |
| PMGC_2481 | CAAAAGAAGGGTAGAGTCTAC | TTCTTCGGTGTGTGTTATTGC | Heterozygous | Heterozygous |
| PMGC_433 | GCAGCATTGTAGAATAATAAAAG | AAGGGGTCTATTATCCACG | Heterozygous | Heterozygous |
| ORPM_214 | TTTTCACAAGCCTCGAAGGA | TGGAAGACCCGAACTTTTTC | Heterozygous | Heterozygous |
| PMGC_333 | CTTAGTGGTGAAGTATTC | GAGTGGGTGCTGATTCATCC | Homozygous | Heterozygous |
| PMGC_2765 | GAGATAGCATCACCTAGAGG | GATATGTCAAGGAAATCCTTAG | Heterozygous | Heterozygous |
| PMGC_2866 | ATTGTTCAAAATCCTCAGGTTC | TAGCATAGTAGCTAGCTAGTG | Heterozygous | Heterozygous |
| ORPM_410 | TGCTTTCCATTCCTTTGCTT | TGAAGTCTTGTGAGCTGAAAGTG | Heterozygous | Homozygous |
| PMGC_1349 | CTGGCATTATAGGTGCTCTC | GGGACTCATTCAAGTTCAGA | Heterozygous | Heterozygous |
| GCPM_250 | ATTTGCAGCTTTAGTGCTTT | TCAAAGGTACGTGATTTTCC | Homozygous | Heterozygous |
| PMGC_2839 | AACCCATAGCAAGAAGCTAG | CAATTACCGAAGAGGATTACTG | Heterozygous | Heterozygous |
| ORPM_1295 | CCTTAGCCTTCTCACACAAC | GGTCTCCATTTTAGCTGTCA | Heterozygous | Heterozygous |
