# Supplementary material for: Genetic Map Construction and Detection of Genetic Loci Underlying Segregation Distortion in an Intraspecific Cross of Populus deltoides
Source: PLoS One. 2015 May 5;10(5):e0126077. doi: 10.1371/journal.pone.0126077 (PMC4420497; doi:10.1371/journal.pone.0126077)
Supplement: S3 Table — Note: “*” indicates significance at α = 0.05; “**” indicates significance at α = 0.01; the probabilities for clustering or dispersal of AFLP markers(mi≥λ i or mi≤λ i) were evaluated by using a two-tailed cumulative Poisson calculator (P ≤ 0.025 is significant at α = 0.05). (DOCX) [file pone.0126077.s004.docx]

Table S3.

| **Linkage group** | **The observed map Length (cM)** | **The expected**  **map Length**  **(cM)** | **The expected number of AFLPs** | **The observed number of AFLPs** | **Poisson two-tailed *P*-value** |
| --- | --- | --- | --- | --- | --- |
| I | 224.2 | 229.35 | 64.56 | 88 | 0.0032** |
| II | 121.2 | 126.97 | 35.74 | 43 | 0.1304 |
| III | 130.2 | 136.12 | 38.32 | 45 | 0.1587 |
| IV | 128.6 | 135.37 | 38.11 | 39 | 0.4638 |
| V | 58.4 | 63.07 | 17.75 | 26 | 0.0391 |
| VI | 59.5 | 70.32 | 19.79 | 12 | 0.0428 |
| VII | 93 | 98.17 | 27.63 | 37 | 0.0508 |
| VIII | 56.1 | 63.58 | 17.9 | 16 | 0.3842 |
| IX | 111 | 119.54 | 33.65 | 27 | 0.1434 |
| X | 116.2 | 121.14 | 34.1 | 48 | 0.0142* |
| XI | 140.2 | 149.25 | 42.01 | 32 | 0.0665 |
| XII | 142.9 | 157.94 | 44.46 | 20 | 0.0000** |
| XIII | 74 | 84.57 | 23.81 | 15 | 0.0373 |
| XIV | 81.3 | 88.69 | 24.97 | 23 | 0.3965 |
| XV | 90.4 | 99.01 | 27.87 | 22 | 0.1538 |
| XVI | 82.5 | 91.67 | 25.8 | 19 | 0.1033 |
| XVII | 80.1 | 89 | 25.05 | 19 | 0.1314 |
| XVIII | 100.5 | 107.68 | 30.31 | 29 | 0.4532 |
| XIX | 50 | 53.85 | 15.16 | 27 | 0.0038** |
| Total | 1940.3 | 2085.28 | 587 | 587 |  |
