# Supplementary material for: Genetic Map Construction and Detection of Genetic Loci Underlying Segregation Distortion in an Intraspecific Cross of Populus deltoides
Source: PLoS One. 2015 May 5;10(5):e0126077. doi: 10.1371/journal.pone.0126077 (PMC4420497; doi:10.1371/journal.pone.0126077)
Supplement: S4 Table — (DOCX) [file pone.0126077.s005.docx]

Table S4.

| **Linkage group** | **Number of markers** | **Number of distorted markers** | **Percentage**  **(%)** |
| --- | --- | --- | --- |
| I | 65 | 20 | 30.77 |
| II | 21 | 0 | 0 |
| III | 18 | 2 | 11.11 |
| IV | 33 | 0 | 0 |
| V | 37 | 0 | 0 |
| VI | 33 | 0 | 0 |
| VII | 35 | 0 | 0 |
| VIII | 31 | 0 | 0 |
| IX | 30 | 16 | 53.33 |
| X | 29 | 17 | 58.62 |
| XI | 21 | 0 | 0 |
| XII | 17 | 6 | 35.29 |
| XIII | 28 | 0 | 0 |
| XIV | 16 | 7 | 43.75 |
| XV | 21 | 0 | 0 |
| XVI | 34 | 1 | 2.94 |
| XVII | 15 | 3 | 20 |
| XVIII | 20 | 1 | 5 |
| XIX | 15 | 0 | 0 |
| Genome wide | 519 | 73 | 14.07 |
