# Supplementary material for: Genetic Map Construction and Detection of Genetic Loci Underlying Segregation Distortion in an Intraspecific Cross of Populus deltoides
Source: PLoS One. 2015 May 5;10(5):e0126077. doi: 10.1371/journal.pone.0126077 (PMC4420497; doi:10.1371/journal.pone.0126077)
Supplement: S5 Table — (DOCX) [file pone.0126077.s006.docx]

Table S5.

| **Linkage group** | **Number of markers** | **Number of distorted markers** | **Percentage**  **(%)** |
| --- | --- | --- | --- |
| I | 88 | 2 | 2.27 |
| II | 45 | 1 | 2.22 |
| III | 46 | 12 | 26.09 |
| IV | 39 | 1 | 2.56 |
| V | 26 | 1 | 3.85 |
| VI | 12 | 0 | 0 |
| VII | 38 | 0 | 0 |
| VIII | 17 | 0 | 0 |
| IX | 28 | 0 | 0 |
| X | 48 | 2 | 4.17 |
| XI | 33 | 2 | 6.06 |
| XII | 20 | 0 | 0 |
| XIII | 15 | 0 | 0 |
| XIV | 23 | 0 | 0 |
| XV | 22 | 2 | 9.09 |
| XVI | 21 | 3 | 14.29 |
| XVII | 20 | 1 | 5 |
| XVIII | 29 | 0 | 0 |
| XIX | 27 | 1 | 3.7 |
| Genome wide | 597 | 28 | 4.69 |
